# Patients’ & students’ perspectives on bedside teaching: A descriptive study

**DOI:** 10.12669/pjms.40.10.8743

**Published:** 2024-11

**Authors:** Farhat Rehana Malik, Farzeen Khan

**Affiliations:** 1 Dr. Farhat Rehana Malik, MBBS, MPH, CHPE, PGD. Department of Community Health Sciences, Peshawar Medical College, Warsak Road, Peshawar, Paksitan. Riphah International University, Islamabad, Pakistan; 2 Dr. Farzeen Khan, BDS, MPH, M.Sc. Department of Community Dentistry, Peshawar Dental College, Warsak Road, Peshawar, Pakistan. Riphah International University, Islamabad, Pakistan

**Keywords:** Anxiety, Communication, Medical Students, Patients, Barriers, Teaching Methods

## Abstract

**Objective:**

To assess the student’s and patients’ perspectives about bedside teaching and to identify possible barriers that delay effective bedside teaching.

**Methods:**

This cross-sectional study was conducted at two teaching hospitals in Peshawar with 153 participants through non-probability serial sampling from December 2017 to March 2018. Interview-based validated questionnaires were used and pilot tested as well. Ethical approval was taken with the participant’s consent. Data were analyzed using SPSS version 19.0 using descriptive statistics.

**Results:**

Among the total 79 students, all of them completed the questionnaires with a 100% response rate. However, from the 76 patients only 74 responded with a response rate of 97%. The majority of the patients (n=58; 78%) were satisfied with bedside teaching with no problems faced. During the bedside teaching only (n= 13; 18.2%) felt anxious while others, enjoyed (n= 59; 80.5%) with satisfaction and only 13% (n= 10) thought their privacy was breached. The satisfied students with the time spent on bedside teaching were (n= 52; 65.5%) while (n= 30; 38.7%) thought thirty minutes time was not enough. The hurdles faced were lack of practice (37.3%), fear of embarrassment in front of peers (21.3%), and lack of confidence to approach the patient (16%).

**Conclusion:**

The patients showed positive attitude and enjoyed bedside teaching. The students preferred it as a valuable tool for clinical experience. However, time constraints, lack of practice, lack of confidence, confidentiality, and anxiety must be overcome to make bedside teaching a useful method. Effective teaching still requires good teaching methodologies with confidence and competency.

## INTRODUCTION

Bedside teaching is a form of small group discussion at the bedside of patient.^1^ WHO (World Health Organization) Advisory Committee on Medical Training suggested in 1992 ‘’bedside teaching should be incremented in the teacher-student and patient-student relationship’’.^2^ Clinical skills associated to physician-patient communication, physical examination, clinical reasoning and professionalism are enhanced at the bedside.^3^ Bedside teaching provided a platform where senior physicians could pass their skills to young intellects, a perfect channel for the flow of knowledge and experience. Motivated students participate in clinical reasoning and solving problem with adequate demonstration and guidance.^2^,^4^,^5^ Bedside teaching is the first step towards clinical environment which needs to be polished to perfection because it lays the foundation of adapting to practice in the future. Despite this, only 48% students reported adequate time for bedside learning^2^ and studies showed bedside teaching as 100% effective tool for learning clinical skills. There was no standard time allotted to bedside teaching, but average time was from 8 to 12 hours per week which did not suit the needs of the students.^6^ Most students (73.3%) felt comfortable in bedside teaching delivered by a junior doctor rather consultants.^7^ Patients being an inherent part of bedside teaching, the case presentations at bedside support physicians to observe patients as real people rather than disease host. In the study, 87% patients said that at bedside discussion they had not been upset.^8^ In a study 50% of the patients found it confusing when medical terminologies are being used during discussions.^9^ Barriers have been pointed out in other studies. New technology, increased workloads and the negligence in effective bedside teaching and lack of intellectual excitement has compromised the clinical skills of young doctors.^10^

In a qualitative study, patients had a submissive role in medical education to impart knowledge to the medical students. Patients comforted themselves in the knowledge that they were following Allah’s command when they were involved in the teaching of medical students.^11^ Bedside teaching “has been neglected and rendered haphazard, mediocre and lacking in intellectual excitement, so much so that the clinical examination skills of young doctors have been seriously compromised” as quoted in research paper they evaluated the research tool upon the medical students for bed side teaching with 0.9627 Cronbach’s Alpha proved to be reliable .^12^

All the participants in this study thought that teaching management of patients was the part best covered by ward rounds. When assessing an ideal ward round, the highest mean rating was for teaching of management of patients, followed by bedside examination and then clinical skills teaching.^13^

A descriptive study in relation to various teaching methodologies was conducted with feedback to have a drift from typical learning methodologies to modern ones with inclusion of videos. Majority of students preferred bedside teaching in real time situations.^14^ A qualitative and quantitative study was carried upon experienced consultants with their perceptions regarding teaching the clinical students by the patient’s bedside. This study proposed and highlighted the gaps to effective teaching and learning. The facts put forward were, no planned authentic curriculum for bedside teaching, lack of responsibility & commitment from the learner’s side with low salary packages of the teachers that mostly affected this discipline.^15^ Another study explored the issues and perceptions towards bedside teaching. The concluding factors responsible for effective bedside teaching came out to be as cordial and conducive environment for learning, student’s commitment, professional behaviour, too many patients, time constraint and lack of significance and understanding bedside teaching as a discipline.^16^

Bedside teaching (BST) is highly under- researched area especially in Pakistan. Very few studies have been done on such an important modality of medical education. There have been studies into the attitudes of the patients regarding bedside teaching, but limited literature is available on bedside teaching in Pakistan and from abroad, though very old articles. There was scarcity of data in relation to this important issue. This paved the way for the conduction of such research to provide us for some basis, to assess patient’s/student’s perspective and identify the possible barriers that delay the effective learning process.

## METHODS

This cross-sectional study was conducted in Peshawar from December 2017 to March 2018. Sample size was calculated using G power software version 3.1.9.2 as 153. The calculated sample size was further divided into 76 patients and 79 students. Only 74 (97%) student’s response rate. However, all the students completed the questionnaires with 100% response rate.

### Ethical Approval:

The study was approved by the Ethical Review Committee of the Quaid-i-Azam University (Ref# DAS/19; July 3, 2019).

A total of 79 medical students and 76 admitted patients in Medical and Surgical wards were chosen through purposive sampling. Admitted patients who had experienced bedside teaching were included in the study and 4^th^ and final year MBBS (Bachelors of Medicine and Surgery) students were involved. The outpatients and inpatients not involved in BST were excluded. Pre-clinical & 3^rd^ year MBBS students were also excluded as they have less exposure to bedside teaching.

There were two set of questionnaires used for students and the other for the patients. Both the questionnaires were validated self-administered tools.^1^,^15^ The patient’s questionnaire had eighteen questions with four domains like;

- Socio-demographic details of age, gender, educational level,
- Perspectives regarding acceptance of students,
- Impressions of bedside teaching and
- Acceptance of bedside teaching & examination from the medical students.

Student’s questionnaires included eleven questions like socio-demographics of age, gender and year of study. Rest of the domains included agreement of with BST, the environment around, burden of the teaching, agreement of patients for students teaching to them, appreciation of the patients towards the teaching, is there any risk of increased infectious diseases, student’s perception of hurdles in BST and different techniques of BST.

After taking written informed consent, data was collected through interviews by a team of researchers. The researchers were trained by their supervisors in taking questionnaire-based interviews in two groups from the students and patients. The collected data was analyzed using SPSS Version-19 and descriptive statistics were calculated in the form of frequency and percentages.

### Consent:

Consent for publication was also taken from all the participants with confidentiality ensured.

## RESULTS

For patients’ perspectives, a total of 76 patients were interviewed based on questionnaires, amongst which 74 responded. The socio-demographic variables of the patients were age (less than 40, more than 40), gender (male and female), education level (primary level or under, secondary or higher). Almost 50.6% of the patients were in the age group of 40 years or less while the remaining 49.4% were 40 years or older and 40.3% of the patients were males and 59.7% were female. Amongst the patients interviewed, 84.4% had received only up to a primary education and 15.6% had received a secondary education.

A total of 79 students (4^th^ year and final year) were given questionnaires that they filled and amongst them 28% were male and 72% were female. Amongst the 75 students, 56% were 4th year students and 44% were final year students, who had had more experience with bedside teaching. The impression of patients about bedside teaching was remarkable. The 68.8% better understood their medical condition. However, 80.5% of the patients enjoyed bedside teaching and only few (18.2%) felt anxious and even less (13%) thought that their confidentiality was breached with bedside sessions. And 89.9% responded that the discussion at bedside teaching was not inappropriate. When asked about disease exposure, 77.3% of the students believed a significantly increase whereas 22.7% thought this was not so. The 38.7% of the students reported up to 30 minutes on average spent on bedside teaching, whereas 33.3% stated up to one hour was spent while, 10.7% students said that two hours on average was given to bedside teaching. A 6.7% stated that more than two hours were given. Amongst these students, 65.5% were satisfied with the time spent on bedside teaching and 34.7% were at a disagreement stating that this time was not sufficient for learning.

**Fig.1:**
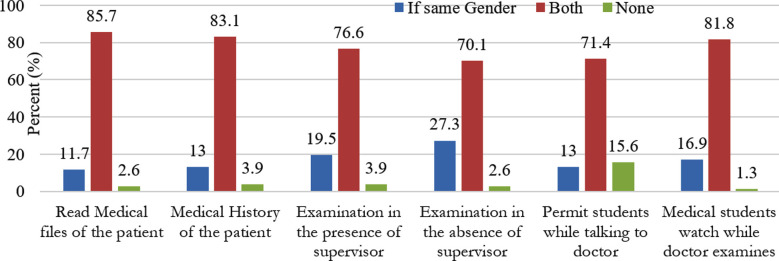
Patients Acceptance of Medical Students.

Patients’ acceptance of medical students: This part of the patients’ questionnaire assessed patients’ acceptance of medical students to view their medical files, take their medical history, examine them in the presence/absence of supervisor, and permit students while consulting their doctor and to allow medical students to watch while the doctor examines them. Overall, the responses of the patients in all categories were positive to higher percentages but 15.6% of the patients preferred to be alone while consulting their doctor’s allowing no students and 16.9% allowed observance by the same gender of their examination by the doctor as shown by Fig.1.

### Medical Files:

Table-I shows that among all the patients interviewed, 85.7% allowed both male and female students to view their medical files, 10.4% preferred only female students to read their files, 1.3% allowed only males and 2.6% did not allow students to view their files at all and preferred professional doctors to read their files.

**Table-I:** Should students view medical files.

Should students view medical files?	Frequency (n)	Percent (%)
Permit male students only	1	1.3
Permit female students only	8	10.1
Permit both genders	66	83.5
Permit none of the genders	2	2.5
Total	77	97.5

### Patients’ impression of bedside teaching:

This part of the questionnaire assessed the impression patients had about bedside teaching. Almost 68.8% of the patients better understood their medical condition after bedside teaching. And 80.5% of the patents enjoyed bedside teaching and only few (18.2%) felt anxious and even less (13%) thought that their confidentiality was breached with bedside sessions but 89.9% responded that the discussion at bedside teaching was not inappropriate.

### Importance of bedside teaching for aspiring doctors according to the patient:

Table-II gives all the details, with 87% of the patients thought it was very important for aspiring doctors whereas 11.7% thought it was not that important. 1.3% stated that it was completely unnecessary.

**Table-II:** Importance of Bedside Teaching.

Importance of Bedside Teaching				

	Frequency	Percent	Valid Percent	Cumulative Percent
Important	67	84.8	87.0	87.0
Un-Important	9	11.4	11.7	98.7
Un-Necessary	1	1.3	1.3	100.0

### Students’ Perspectives:

A total of 79 students (4^th^ year and final year) were given questionnaires that they filled and amongst them 28% were male and 72% were female. Amongst the 75 students, 56% were 4th year students and 44% were final year students, who had had more experience with bedside teaching.

### Time spent on bedside teaching:

The 38.7% of the students reported that up to 30 minutes on average was spent on bedside teaching, whereas 33.3% stated up to one hour was spent while, 10.7% students said that two hours on average was given to bedside teaching. A 6.7% stated that more than two hours were given. Amongst these students, 65.5% were satisfied with the time spent on bedside teaching and 34.7% were at a disagreement stating that this time was not sufficient for learning.

### Skills improved at bedside:

**Fig.2:**
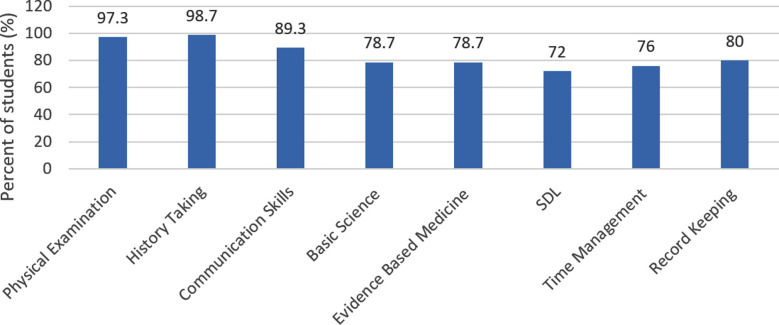
Skills Improved at Bedside Teaching *Note:* SDL: Student Directed Learning.

Almost 100% of the students agreed that physical examination was best improved at bedside. All other skills were also improved. The least improved skill was management of time. This is quite evident from Fig.2.

### Disease exposure:

When asked about disease exposure, 77.3% of the students believed that the exposure to diseases was significantly increased whereas 22.7% thought this was not so.

### Difficulties faced by the students:

When questioned about hurdles and limitations faced during their bedside experiences, 37.3% stated that lack of practice was the main challenge faced, and 21.3% feared embarrassment in front of peers. Moreover, 16% didn’t have the confidence to approach the patient an 13.3% feared they might harm the patient due to inexperience while 8% had a problem overcoming intimacy boundaries and 4% stated previous bad experiences as the main obstacle hindering their progress.

## DISCUSSION

Among the patients, 68.8% better understood their medical condition, 80.5% enjoyed bedside teaching, 18.2% felt anxious however, 13% thought breech of their confidentiality. It was considered appropriate by 89.9%. The average time spent on bedside teaching was reported by 38.7% of the students as 30 minutes, 33.3% stated one hour, 10.7% students said two hours however, 6.7% said more than two hours were given. Among all the patients interviewed, 85.7% allowed both male and female students to view their medical files, 10.4% preferred only females, 1.3% only males. Amongst these students, 65.5% were satisfied with the time spent on bedside teaching and 34.7% disagreed with this time as not sufficient for learning. Further students claimed 97.3%, 98.7% and 89.3% improvement in physical examination, history taking and communication skills respectively due to bedside teaching. In relation to problems faced by the students, 37.3% stated lack of practice, 21.3% feared embarrassment in front of peers. Moreover, 16% had no confidence and 13.3% feared of harm and 4% had bad past experiences.

Most of the patients favored bed side teaching even though most had primary education level. This was in contrast to the studies reviewed in literature. Patients’ permissiveness to BST depended on their education level. Well educated patients were more permissive.^3^ A high percentage of patients 85.7% allowed to read their medical records. Patients’ views about history taking and examination were also positive and 83.1% allowed examination by both genders. However, 13% preferred examination by the same gender. In this study 87% of patients were satisfied with maintenance of confidentiality. One study reported only 18.2% of patients anxious during BST and 81% remained calm while another study warned clinicians not to increase dose of hypertensive drugs in teaching hospitals^4^ as the patients going through bedside teaching can cause rise in blood pressure due to anxiety. The 68% patients believed a better understanding of their disease despite the high number (81%) of patients being uneducated or having education up to primary level. It coincided with the study and that found patients enjoy bedside teaching as they get a better understanding of their illness. All the students agreed that bed side teaching is an efficacious mode of learning the basic skills. Student’s satisfaction with the time spent on BST was fair i.e. 65% which is in conflict with a study that report only 48% of the students think that they receive adequate time at bed side. The time spent on BST varies and it depends on different factors which this study did not investigate and studies should be conducted to find out the factors. Daily 30 minutes to one hour time was considered to be sufficient by the students.^5^

Another study also suggests that average time for BST was 8-12 hours per week and was unsatisfactory. The main limitation to the use of BST is lack of practice (37.3%) according to this study. Other hurdles include fear of embarrassment in front of peers (21%) and 16% stated that don’t have enough confidence to approach the patients and 13.3% feared that they might harm the patient. 6 Innovations in medical education always necessary to bridge the missing links among expectation from the students and actual their practice and learning. A study has reported that better educated were more satisfied than less educated patients and the reason in particular was that they did not find the terminology that has been used confusing.^9^ However, this study showed that despite low education level, these patients were more receptive and welcomed bedside teaching.

A study was done on 143 internal medicine trainees, with a structured self- designed questionnaire. Bed side teaching and clinical skills received the lowest scores by the post graduate trainees as compared with the undergraduate medical students with significant p value (0.001). Patient management received the best scores as quality services were being provided. The maximum number of students (87%) preferred bed side teaching and time allocated was found to be adequate.^13^

A descriptive Pakistani study was conducted upon hundred students to evaluate effective learning methods among them. The academic effective methods in theory were problem-based learning (62%), videos (46%) and power point presentations (41%). The preferred clinical methods came out to be as bedside teaching (44%), mannequins (31%), and audio-visual aids as 25% 14. These results are not in accordance with the present study, as they probed into effective methods of learning and the present one was in relation to one method only.

A Pakistani study assessed the perceptions of clinical students of teaching learning methods with a feedback proforma. The students feedback preferred modern methods of teaching and problem-based learning. A Mix Method study done in Ayub Teaching Hospital, distributed close ended tool to twenty Professors to evaluate their perspectives regarding bedside teaching. Qualitative and quantitative components were recorded. The study concluded curriculum development for bed side teaching with integration from preclinical to clinical years of teaching, environment should be made friendlier and annual confidential report of faculty members should be performance based. The salary should be enhanced, job satisfaction should be assured and private clinical practice for faculty members should be banned so that they could concentrate on their professional responsibilities. The time span suggested came out to be 2- 4 hours. The findings go hand in hand with the present study, however salary was not taken into account in this one 15.

A study by Hussain found an agreement upon cordial relationship with the students, motivation, communication, professional behavior, rush of patient, time constraint as barrier, lack of awareness, use of technology as compared to disagreement. Statistically significant difference was found regarding noisy environment, chance to answer the question, inconvenient time, priority of exam preparation, rush of patient, time constraints, lack of awareness, technology preference than real patients. All these results are in correlation with the present study 16. The findings are similar as the present study results.

The researchers distributed questionnaires to 463 residency programs, for the evaluation of bedside teaching. Only 221 responded and showed 15% teaching in the conference rooms alone whereas 77% at bedside as well as conference room.^17^ The findings suggested teaching away from the beds.^18^ These results are not comparable with the present study. A review article by Dewey JJ et al 19 discussed the effectiveness of bedside teaching with a proposed framework and example was taken from working with patients from neurology department. This article is quite different from the present study as it was done in neurology ward where serious patients are being treated. Moreover, the present study was a quantitative one. An objective study to determine the effectiveness of bedside teaching was conducted upon cardiac patients and results were encouraging with ample amount of learning experience on the part of the students.^20^ This study is comparable with the present study findings.

A study used two groups of medical under-graduates of 7^th^ semester and placed them in medicine ward in their study with conventional method of learning in one group and interventional method in the other. Pre and post-tests assessed the cognitive portion on day one and the last day with psychomotor skills also evaluated among the students involved in the bedside teaching. The group with the novel intervention showed remarkable results, with statistical significance. Positive feedback was received at the end from the undergraduate medical students.^21^

A study was conducted at Oman with usual method of teaching as bedside and as a provision of clinical practice in Canada. The research instrument used was 47- item questionnaire piloted on group of ten students. They were asked to fill that through five-point Likert scale and answers were analyzed by factor analysis. Enhanced learning experience was noted at the end.^22^ The results of these both studies were not in correlation with the present study as methods of analysis were different and two groups were used which were missing in the present study.

### Limitations:

It was a quantitative study with not a large sample size so the actual picture cannot be attained. The results were obtained from a single Tertiary Care Hospital and no comparisons made with other hospitals so these results cannot be generalized. Recall bias could have been introduced as it was concerned with the questionnaires.

## CONCLUSION

The patients showed positive attitude and enjoyed bedside teaching. The students preferred it as a valuable tool for clinical experience. However, time constraints, lack of practice, lack of confidence, confidentiality, and anxiety must be overcome to make bedside teaching a useful method. Effective teaching still requires good teaching methodologies with confidence and competency. Moreover, bedside teaching should be structured with proper guidelines indicated in medical curriculum to have benefits for the patients as well as students.

### Authors’ Contribution:

**FRM & FK:** Conceived, designed, literature search, and did the statistical analysis, prepared the manuscript, editing of the manuscript, Review and final approval of the manuscript, responsible for the intellectual content and integrity of the research.
